# Can Single Buccal Infiltration With 4% Articaine Induce Sufficient Analgesia for the Extraction of Maxillary Teeth? A Systematic Literature Review

**DOI:** 10.7759/cureus.42975

**Published:** 2023-08-05

**Authors:** Abdulelah S Alsager, Hussain M ALGUBEAL, Abdullah F Alanazi, Ahmad Al-Omar

**Affiliations:** 1 Dentistry, College of Dentistry, King Saud University, Riyadh, SAU; 2 Surgery, King Saud University, Riyadh, SAU

**Keywords:** anesthesia, dental, infiltration, articaine, buccal

## Abstract

This systematic review evaluates the efficacy of single buccal infiltration of articaine for extracting upper teeth. A search of the PubMed, Ovid SP, Scopus, Embase, and Cochrane databases for English-language studies published between 2000 and 2021 was performed on August 26, 2022, based on the pre-specified question using the MeSH terms [(buccal) and (articaine) and (infiltration) and (dental)]. Of the 16 clinical trials identified involving 1,339 patients, six compared the subjective procedural pain associated with single buccal infiltration of articaine with that of lidocaine, three of which reported reduced pain and the other three greater success in extraction for the articaine group. Four of the 16 studies compared the procedural pain associated with single buccal infiltration of 4% articaine with double (buccal and palatal/lingual) infiltration; two reported insignificant differences between the groups; and the other two reported greater success using buccal and palatal injections. Five of the 16 studies compared the procedural pain associated with single buccal articaine with double buccal and palatal/lingual infiltration of 2% lidocaine and reported insignificant differences. The other of the 16 studies compared the subjective pain associated with single buccal infiltration of 4% articaine 1:100:000 with single buccal infiltration of 4% articaine 1:200:000 and found a statistically significant difference. All of these studies concluded that upper permanent maxillary teeth can be extracted using only a 4% articaine buccal infiltration, but further investigation is necessary to determine whether this approach can replace the gold standard of buccal and palatal infiltration.

## Introduction and background

Pain control is an essential part of healthcare since many therapies and advanced operations would be impossible without profound anesthesia. Despite its shortcomings, local anesthesia remains the most effective, efficient, and safe method of pain management [1]. This form of anesthesia involves, by definition, a loss of sensation in only one part of the body by inhibiting the conduction of painful stimuli to the central nervous system [2]. Articaine is one of the newest local anesthetic agents, having been approved by the Food and Drug Administration (FDA) in April 2000 [1]. Specifically, articaine {methyl 4- methyl-3-[2-(propylamino)-propanoylamino] thiophene-2-carboxylate} is a local anesthetic amino amide. All amino amide local anesthetics (MOU1) contain benzene rings, unlike articaine, which has a thiophene ring. The thiophene ring renders the anesthetic more potent by allowing for greater lipid solubility. Articaine can be inactivated by serum esterase in a rapid process that occurs in the serum. Another slower process takes place in the liver, where the amide linkage undergoes biotransformation. About 90% of the articaine is metabolized through hydrolysis in the blood through the fast process into articainic acid, which is inactive. This acid is then excreted by the kidneys as articainic acid glucuronide. Articainic acid has a longer serum half-life, 64 minutes, whereas that of articaine is 20 minutes [3]. Regarding safety and efficacy, articaine has proved safe for local infiltration or peripheral nerve blocking in dentistry. It has many uses in medicine, being administered as an epidural, ocular, spinal, and regional nerve block or injected intravenously for regional anesthesia [3], and it has been widely used in dental surgery [4].

Articaine was first synthesized in 1969 in Germany; Winther and Nathalang performed the first clinical trials in 1971; and it was approved for clinical use in 1976 under the name carticaine hydrochloride [5]. The duration and perfusion of 2% articaine with 1:200,000 adrenaline are greater than for 2% lidocaine with 1:200,000, producing profound anesthesia for all of the teeth except the mandibular molars [6]. Carticaine was renamed articaine in 1984 and approved by the USFDA in 2000 as a 4% formula with 1:100,000 epinephrine under the name Septocaine (Septodont), and 4% articaine with 1:200,00 adrenaline was approved by the FDA in 2006 [5]. Local anesthetics are, in general, safe agents [7], and articaine is considered one of the safest because its rapid metabolism into an inactive metabolite minimizes the potential for overdose and systemic toxicity, even after many injections [8]. However, paresthesia, the abnormal sensation or prolonged duration of anesthetic action, may occur temporarily or permanently [7]. Thus, a study of 1,325 individuals who received either lidocaine or articaine injections during dental treatment and later took part in phone interviews identified 53 who reported paresthesia, with a higher percentage for those who received articaine (1 in 49) compared with those who received lidocaine (1 in 63) [9]. A retrospective study of complaints after such injections conducted in Canada in 1995 found a higher frequency of prolonged anesthesia after articaine was used [10].

The other reported adverse effects of articaine include hypersensitivity reactions [11], ophthalmologic complications [12], ischemia of the skin [13], and fever [14]. Regarding extraction, many studies have found articaine to be more efficacious than lidocaine, with 1.5 times the potency and longer duration [15]. Likewise, the onset time of 4% articaine is significantly less than that of 4% lidocaine [16]. Generally, infiltration serves to anesthetize the maxillary teeth, while nerve blocking is done for mandibular teeth using a 2% local anesthetic agent. Because of the high failure rate of the interalveolar nerve block (IANB) and the large amount of local anesthetic solution delivered to the patient, some clinicians use buccal infiltration of articaine for the mandibular posterior teeth in order to overcome these problems. This technique can be more effective than inferior alveolar nerve block, and many studies have been performed to compare 2% lidocaine with 4% articaine for buccal infiltration of mandibular teeth. Thus, a review by Meechan [17] shows that 2% lidocaine is inferior to 4% articaine for this purpose, while Brandt et al. [18], in a review of 13 controlled clinical trials, reported no significant difference in the efficacy of 2% lidocaine and 4% articaine for IANB but found articaine to have a higher success rate than lidocaine after infiltration [18]. The efficacy of articaine for buccal infiltration of mandibular teeth is thought to be greater when it is applied in adequate amounts of local anesthesia; thus, a study by El-Kholey [19] showed 3.6 ml of articaine to have a significantly higher success rate than 1.8 ml (93% and 53%, respectively). This systematic review aims to determine if palatal infiltration can be excluded when single buccal infiltration is given with 4% articaine for the extraction of permanent maxillary teeth.

## Review

Methods 

Protocol

This systematic review is currently registered in the International Prospective Register of Systematic Review (PROSPERO) (ID: CRD42022371728). It follows the preferred reporting items for systematic reviews and meta-analyses (PRISMA) guidelines for reporting.

Search Strategy

The search strategy was designed based on the population intervention comparison outcome (PICO) framework to address the question “Can single buccal infiltration of 4% articaine induce sufficient analgesia for the extraction of maxillary teeth?” The PICO is broken down as follows population (P): adults, the intervention (I): buccal infiltration of articaine, comparison (C): buccal and palatal infiltration of lidocaine, and the outcome of interest (O): anesthesia for the extraction of permanent maxillary teeth.

An electronic search was performed on the PubMed, Ovid SP, Scopus, Embase, and Cochrane databases for English-language studies published between 2000 and 2021 and was completed on August 26, 2022. The search was based on a pre-specified question using the relevant MeSH terms [(buccal) and (articaine) and (infiltration) and (dental)].

Eligibility Criteria

**Figure 1:**
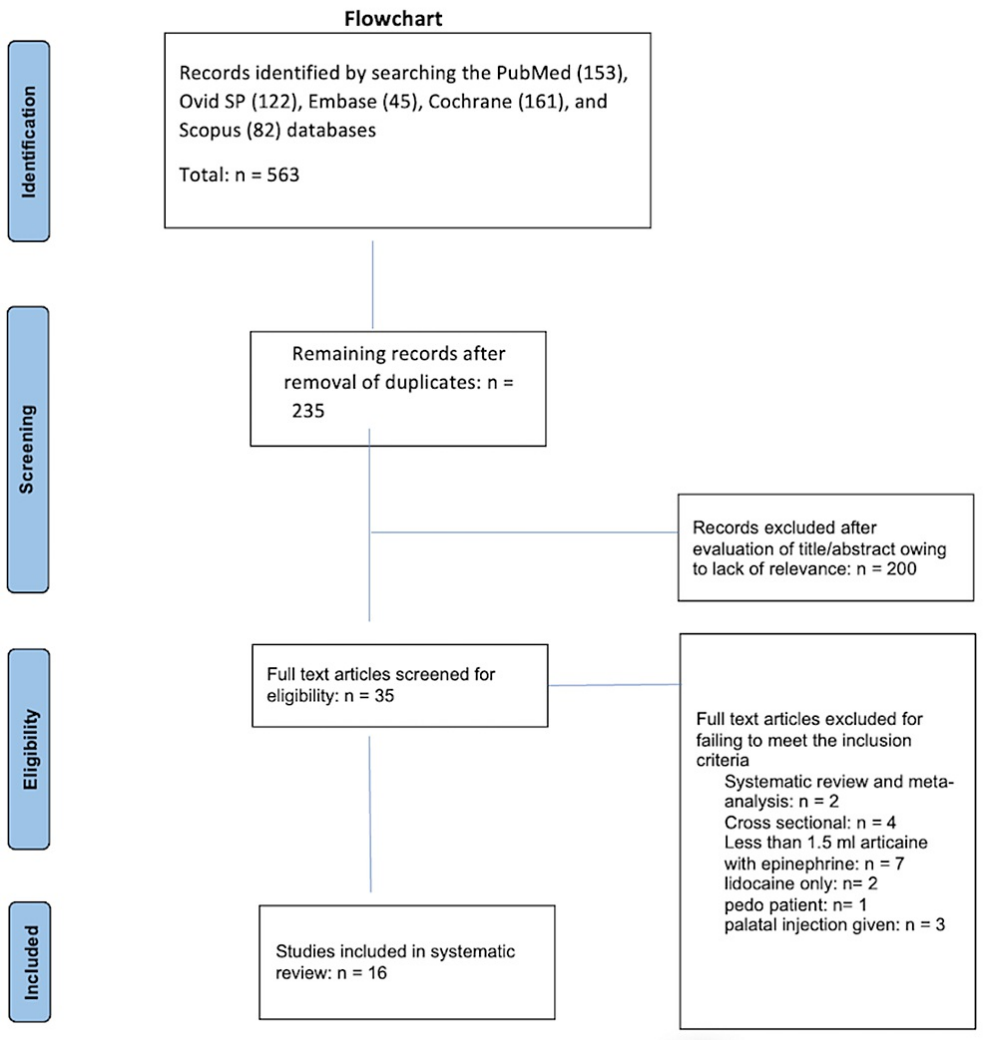
PRISMA 2009 flow diagram

The evaluation included all of the clinical trials that have assessed the success rate of single buccal infiltration with more than 1.5 ml of 4% articaine and/or compared it with a single buccal or standard buccal and palatal injection of 2% lidocaine in terms of inducing sufficient anesthesia to extract upper teeth from adults. The preliminary studies were retrieved using the MeSH terms from the databases. All of the duplicates were then excluded and the titles and abstracts were screened. Two reviewers evaluated the full texts of potentially relevant studies and recorded the authors’ names, the year of publication, the country in which the research was conducted, the study design, the characteristics of the sample, the age of the participants, the nature of the intervention, the nature of the comparison, the pain scale used, and the conclusions reached on a Microsoft Excel sheet. The outcome of interest was “pain during extraction when using a single buccal infiltration with 4% articaine.” Sixteen articles met the eligibility criteria in this review (Figure 1).

Risk-of-Bias (RoB) Assessment

Four members of the review team used the Cochrane Collaboration criteria [20] to evaluate seven parameters independently, including random sequence generation, the concealment of allocation, the blinding of the subjects (participants and personnel), the blinding of the evaluator (the individual assessing the outcome), the completeness of the outcome data, the selective reporting of outcomes, and bias owing to other sources. The bias ratings for this study were designated “high,” “unclear,” and “low.” Thus, the parameters with a high risk of bias for a given study were categorized as such; an unclear risk of bias was identified in studies with one or more unclear parameters; and the studies with a low risk of bias for all seven parameters were also categorized as such. In this way, each of the included studies was classified separately as having either a low, unclear, or high overall risk of bias by the four reviewers, who, after comparing the scores, reached a consensus on the final decision.

Results

The initial search using the MeSH terms revealed 563 articles, of which 328 were duplicates. The titles and abstracts of the 235 articles remaining after the removal of the duplicates were screened. The full texts of the 35 potentially relevant papers thus identified were then evaluated [21-55], and 19 of them were excluded (22, 24-27, 30, 32-34, 39-42, 44, 49-50, 52 and 54-55) for the reasons presented in Table 1. The remaining 16 served as the sample for this final systematic review (21, 23, 28-29, 31, 35-38, 43, 45-48, 51 and 53).

**Table 1:** Studies excluded from the review

Number	Excluded article	Reason for exclusion
1	Majid and Ahmed 2017 [25]	palatal injection was given as a placebo
2	Badenoch-Jones et al. 2017 [26]	cross-sectional study (survey)
3	Badenoch-Jones et al. 2016 [27]	systematic review
4	Hassan et al. 2011 [32]	less than 1.5 ml articaine administered
5	Sharma et al. 2014 [33]	less than 1.5 ml articaine administered
6	Badcock 2007 [34]	cross-sectional study (survey)
7	Khan and Qazi 2017 [40]	only lidocaine used
8	Bahrololoomi and Maghsoudi 2022 [41]	conducted in pediatric clinics
9	Gholami et al. 2021 [42]	less than 1.5 ml articaine administered
10	Cui et al. 2018 [44]	meta-analysis
11	Gazal 2020 [49]	palatal injection of articaine
12	Deshpande et al. 2020 [50]	less than 1.5 ml articaine administered
13	Azad et al. 2019 [52]	less than 1.5 ml articaine administered
14	Joshi and Soni 2019 [54]	less than 1.5 ml articaine administered
15	Shalash and Eladl 2019 [55]	less than 1.5 ml articaine administered
16	Friedl et al. 2012 [22]	less than 1.5 ml articaine administered
17	Lima Jr et al. 2009 [24]	cross-sectional study
18	Sekhar et al. 2011 [30]	only lidocaine used
19	Isik et al. 2011 [39]	cross-sectional study

Characteristics of the Included Studies

The characteristics of the 16 included studies are summarized in Table 2. They were published between 2000 and 2021, involved permanent teeth only, and enrolled participants ranging in age from 12 to 84 years.

**Table 2:** Characteristics of the included studies

No.	Author(s) and year	country	Study design	Sample characteristics	Intervention	Comparison	Topical anesthesia and needle gauge (G)	Extraction	Pain scale	Conclusions
1	Somuri et al. 2012 [21]	India	Randomized single-blinded cross-over.	30 adult patients, 19 women, and 11 men ranging in age from 10 to 30 years, and divided into two groups.	1.7 ml single buccal infiltration of 4% articaine + 1:100,000 adrenaline.	1.75 ml buccal injection+ 0.25 ml palatal injection of lidocaine + 1:100,000 adrenaline.	Not mentioned.	Bilateral maxillary premolar extraction.	visual analog scale (VAS), faces pain scale (FPS) to rate the pain on extraction.	Single buccal infiltration can be sufficient to obtain palatal anesthesia.
2	Lima Jr et al. 2013 [23]	Brazil	Double-blinded controlled clinical.	30 patients ranging in age from 15 to 46 years and divided into two groups.	1.8 ml buccal infiltration of 4% articaine with 1: 100,000 adrenaline.	1.8 ml buccal infiltration of 4% articaine with 1: 200,000 epinephrine.	Not mentioned.	Extraction of a partially impacted upper third molar with pericoronitis.	Hand gestures.	In maxillary third molar with pericoronitis extraction without palatal injection 4% articaine with 1: 100,000 epinephrine, is more effective than 4% articaine with 1: 200,000 epinephrine.
3	Bataineh and Al-Sabri 2016 [28]	Jordan	A prospective controlled study following a split-mouth protocol.	48 patients served as the control; 27 male and 21 female participants ranging in age from 28 to 84 years.	Single buccal injection of 1.8 ml 4% articaine with 1: 100,000 adrenaline.	None.	27 G needle measuring 0.40 X 30 mm	Extraction of anterior and posterior maxillary teeth	Visual analog scale (VAS), verbal rating scale (VRS)	Maxillary anterior and posterior teeth can be extracted with single buccal infiltration when palatal soft tissue manipulation is not required
4	Fan et al. 2009 [29]	China	Randomized controlled trial.	71 patients, 38 men, and 33 women; 142 total teeth were extracted.	Single buccal injection of 1.7 ml 4% articaine HCl with epinephrine 1:100,000.	Identical protocol applied for buccal injection; palatal infiltration of 0.4 mL 4% articaine HCl with epinephrine 1:100,000.	Sterile dental needle, 30 G, 0.3 x 21 mm.	Permanent maxillary tooth removal (33 wisdom teeth, partly or fully erupted, and 41 orthodontic teeth; the rest of 15/32 and 17/32, 14/36, and 22/36 were back and front teeth on the experimental and control sides, respectively).	Visual analog scale VAS).	When using articaine HCl for routine maxillary permanent tooth extraction, palatal injection is possibly not needed.
5	Luqman et al. 2015 [31]	Pakistan	Randomized controlled trial.	194 patients, 113 male, and 81 female, ranging in age from 20 to 60 years.	Single buccal infiltration of 4% articaine with 1:200,000 adrenaline in a cartridge ampule of 1.7 ml (100).	Single buccal infiltration of 2% lidocaine HCl with 1:100,000 adrenaline in a cartridge ampule of 1.8 ml.	Sterile single-use 27 G 0.40 x 21 mm disposable dental needle.	Simple tooth extraction in the maxillary arch from three groups: group 1 (posterior teeth) including the first, second, and third molars on either side; group 2 (middle teeth) including the premolars; group 3 (anterior teeth) including incisors and canines.	Visual analog scale (VAS), face pain scale (FPS).	With 4% articaine as a single buccal injection, maxillary teeth can be extracted without palatal injection.
6	Bataineh et al. 2019 [35]	Jordan	A single-blinded clinical trial with randomization.	155 patients, 51 male, and 104 female, ranging in age from 13 to 62 years.	The experimental group received only a buccal injection of 4% articaine with 0.012 mg/ml epinephrine (one cartridge served as a first buccal injection).	Positive control group received buccal and palatal local anesthetic injections of 2% lidocaine with 0.015 mg/ml epinephrine (three-quarters of a cartridge injected buccally and one-quarter palatally) Negative control group received only buccal local anesthetic injection of 2% lidocaine with 0.015 mg/ml epinephrine (one cartridge used as a first buccal injection).	Not mentioned.	Extraction of permanent maxillary teeth.	Visual analog scale (VAS) and verbal response scale (VRS)	Extraction of maxillary teeth is possible without using palatal injection; no difference was found between articaine and lidocaine.
7	Kumar et al. 2019 [36]	India	A triple-blinded randomized controlled trial.	100 patients, 54 male, and 46 female, ranging in age from 18 to 60 years.	Single buccal infiltration with 1.8 ml articaine HCl. 4% with epinephrine 1:100,000 Injection (50 Patients).	Single buccal infiltration with 1.8 ml lidocaine HCl. 2% and epinephrine 1:100,000 (50 Patients).	Sterile 27 G disposable needles.	Maxillary first molar extraction.	Visual analog scale (VAS).	Maxillary first molar can be extracted without palatal injection; single buccal infiltration can reduce patient pain and has a comparable effect to buccal and palatal injections with lidocaine.
8	Sandilya et al. 2019 [37]	India	A double-blinded randomized clinical trial with a split‐mouth design.	100 patients, 64 male, and 36 female, ranging in age from 12 to 30 years.	Only buccal infiltration (1.75 ml) of 4% articaine with 1:100,000 adrenaline.	Buccal (1.75 ml) and palatal (0.5 ml) infiltration of 2% lidocaine with 1:200,000.	Not mentioned.	Bilateral extractions of permanent noncarious maxillary first or second premolars for orthodontic reasons.	Visual analog scale (VAS).	Articaine. as a single buccal infiltration, can be used as an alternative to lidocaine for the extraction of maxillary premolars.
9	Saravanan et al. 2015 [38]	India	Single-centered, balanced randomized, double-blinded, parallel-group study.	116 patients. 55 male and 61 female, ranging in age from 15 to 65 years.	Administered 1.7 ml of 4% articaine HCl with adrenaline 1:100,000; articaine anesthetic agent was injected into the buccal vestibule by simple infiltration method.	Administered 1.7 ml of lidocaine 2% with adrenaline 1:80,000 in a similar manner.	Not mentioned.	Maxillary teeth that are grossly destroyed by caries, infected root stumps, impacted maxillary third molars, or therapeutic extraction of premolars.	Visual analog scale (VAS).	Bone diffusion of 4% articaine is greater than 2% lidocaine; palatal injection is not absolutely required for extraction of maxillary teeth.
10	Chandrasekaran et al. 2021 [43]	India	Prospective double-blinded randomized control trial.	150 patients, 57 male, and 93 female, ranging in age from 18 to 45 years.	Group A patients were administered 4% articaine local anesthetic with 1:100,000 adrenaline (1.8 ml) as a single buccal infiltration.	Group B patients were administered 0.5% bupivacaine local anesthetic with 1:100000 adrenaline (1.8 ml) as a single buccal infiltration, Group C patients were administered as a local anesthetic 2% lidocaine with 1:100,000 adrenaline (1.8 ml) in a with single buccal infiltration.	Not mentioned.	Requiring extraction of maxillary teeth and mandibular anterior teeth.	Visual analog scale (VAS), facial pain scale (FPS).	Bupivacaine and lidocaine cannot be used as a single buccal injection to anesthetize the palatal tissue but articaine is successful in 98% of cases.
11	Uckan et al. 2006 [45]	Turkey	Controlled clinical trial.	53 patients, 25 female, and 28 male, ranging in age from 18 to 48 years.	Single buccal infiltration of 2 ml articaine 4% epinephrine with 1:100 000 adrenaline.	1.75 mL of articaine was injected into the buccal site with a palatal injection of 0.25 ml.	Not mentioned.	Extraction of permanent maxillary teeth.	Faces pain scale (FPS), visual analog scale (VAS).	Extraction of permanent maxillary teeth is possible with a single buccal injection using 2 ml of articaine.
12	Sochenda et al. 2020 [46]	Thailand	Prospective, clinical crossover experiment, randomized split-mouth controlled trial.	28 patients, 10 male, and 18 female, ranging in age from 18 to 45 years.	Buccal vestibule infiltration of 4% articaine with 1:100,000 epinephrine 1.7 ml injected without palatal infiltration.	Buccal and palatal infiltration of 4% articaine with 1:100,000 epinephrine injected.	Not mentioned.	Maxillary impacted third molar surgery.	Visual analog scale (VAS) and a numeric rating scale.	Single buccal infiltration can be an alternative to the conventional technique for surgical extraction of impacted maxillary third molars.
13	Phyo et al. 2020 [47]	Thailand	A randomized double-blind study.	30 patients, 6 male, and 24 female.	1.7 ml single buccal infiltration of 4% articaine with 1:100,000 epinephrine.	1.7 ml single buccal infiltration of 4% lidocaine with epinephrine 1:100,000.	27 G needle attached to a 3 cc disposable syringe.	Bilateral surgical removal of symmetrically-positioned maxillary third molars.	Visual analog scale (VAS), numerical rating scale (NRS).	Palatal anesthesia can be obtained using a single buccal infiltration with 4% lidocaine and 4% articaine for maxillary third molar surgery depending on the impaction classification.
14	Rayati et al. 2021 [48]	Iran	Double-blinded randomized clinical trial.	139 patients, 65 male, and 74 female, ranging in age from 20 to 60 years.	1.8 ml single buccal infiltration of 2% lidocaine with epinephrine 1:100,000.	1.8 ml single buccal infiltration of 4% articaine with epinephrine 1:100,000.	Short 21 mm 27 G needle.	Extraction of maxillary molars.	Not mentioned.	Depth of anesthesia in palatal tissue with single buccal infiltration using 4% articaine may differ depending on bone thickness and tooth condition.
15	Iyengar et al. 2020 [51]	India	-	50 patients ranging in age from 15 to 40 years.	2 ml single buccal infiltration of 4% articaine with adrenaline 1:100,000.	Buccal and palatal injections of 4% articaine with adrenaline 1:100,000 (1 ml and 0.5 ml).	Not mentioned.	Extraction of bilateral permanent maxillary posterior teeth.	Visual analog scale (VAS), Wong-Baker Facial Pain Scale.	Operators cannot rely on the diffusing capacity of articaine to anesthetize palatal tissue; a single buccal injection is preferable for young patients.
16	Al-Mahalawy and El-Mahallawy 2020 [53]	Egypt	Randomized, controlled, split-mouth clinical trial.	45 patients, 24 male, and 21 female.	Single labial infiltration injection of 1.7 ml of 4% articaine with 1:100,000 adrenaline.	1.5 ml labial infiltration injection followed by 0.3 ml nasopalatine injections of 2% lidocaine with 1:100,000 adrenaline.	27 G short needle.	Extraction of maxillary anterior teeth.	Visual analog scale (VAS).	With a single labial infiltration using 4% articaine, a nasopalatine nerve block may no longer be necessary.

**Figure 2:**
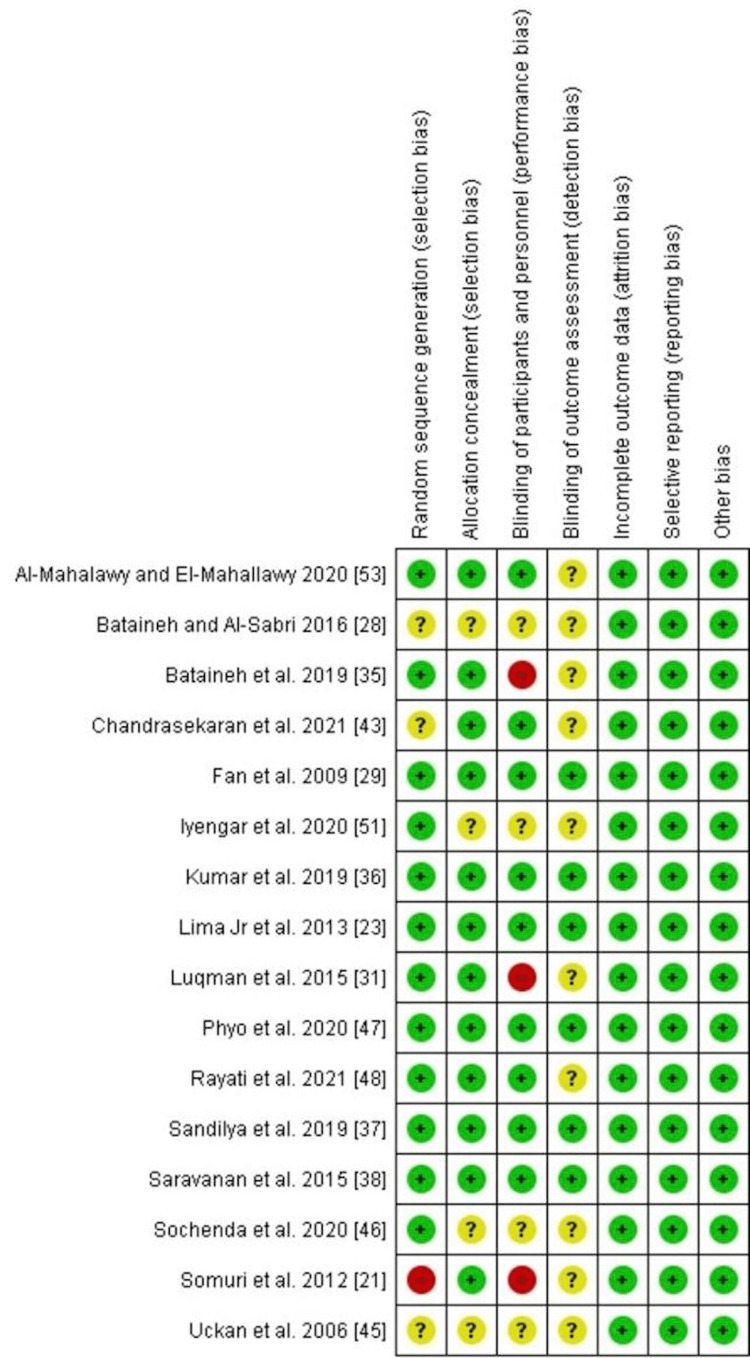
Risk of bias summary

The risk of bias (Figure 2) was evaluated for each study following the Cochrane guidelines [20]. Most of the studies involved randomization [23, 29, 31, 35-38, 46-48, 51, 53], with four exceptions [21, 28, 43, 5]. Most also involved allocation concealment, again with four exceptions [28, 45, 46, 51]. Blinding of the participants was done in more than half of the studies the exceptions being [21, 28, 31, 35, 45-46, 51], while there was no clear blinding of the outcome in more than half the exceptions being [23, 29, 36-38, 47]. None of the studies reported observing attrition bias, reporting bias, or any other bias. 

Discussion

All sixteen studies included in the review were clinical trials. The single-blinded design was followed in the studies by Somuri et al. [21], Fan et al. [29], Luqman et al. [31], Bataineh et al. [35], and Saravanan et al. [38]. The studies by Lima Jr et al. [23], Sandilya et al. [37], Chandrasekaran et al. [43], Phyo et al. [47], Rayati et al. [48], and Al-Mahalawy et al. [53] used a double-blinded design, and that by Kumar et al. [36] used a triple-blinded design. The other studies, by Bataineh et al. [28], Uckan et al. [45], Sochenda et al.[46], and Iyengar et al. [51] did not specify the design. As already noted, the ages of the participants in the included studies ranged from 12 to 84 years.

The studies included in this systematic review except that by Bataineh et al. [28] compared the single buccal infiltration of 4% articaine 1:100:000 with (1) single buccal infiltration of 2% lidocaine, (2) buccal and palatal infiltration of 4% articaine, (3) buccal and palatal infiltration of 2% lidocaine, or (4) single buccal infiltration of 4% articaine 1:200:000. The following discussion addresses each of these treatments in turn as well as (5) the study by Bataineh et al. [28], which included no control group and evaluated only the efficacy of single buccal infiltration of 4% articaine.

Single Buccal Articaine Compared With Single Lidocaine

Five of the studies evaluated procedural pain during the extraction of maxillary teeth for the comparison of single buccal infiltration of 4% articaine with single buccal infiltration of 2% lidocaine Bataineh et al. [35], Kumar et al. [36], Saravanan et al. [38], Chandrasekaran et al. [43], Rayati et al. [48]. In one study by Phyo et al. [47], the comparison was between 4% articaine and 4% lidocaine. The studies evaluated procedural pain during the extraction of upper permanent maxillary teeth except that of Chandrasekaran et al. [43], which included the lower anterior teeth. Five studies - Bataineh et al. [35], Kumar et al. [36], Saravanan et al. [38], Chandrasekaran et al. [43], and Phyo et al. [47] evaluated procedural pain during the extraction using a subjective score, the visual analog scale (VAS), while the sixth Chandrasekaran et al. [43] used an objective score, the facial pain scale (FPS). Bataineh et al. [35] found that 62% of the patients (31) in the lidocaine group reported mild pain and 60% (30) patients in the articaine group while 34% of patients (17) in each group reported moderate pain and 4% (two) of the patients in the lidocaine group and 6% (three) of those in the articaine group reported severe pain. Only two patients in the lidocaine group and three patients in the articaine group required an additional palatal injection. The authors attributed the higher success rate in the lidocaine group to the higher concentration of epinephrine. Kumar et al. [36] distinguished four categories in the perceptions of pain during the extraction, none, mild, moderate, and severe. There were no reports of no or severe pain. Most of the patients in the articaine group (88%) reported mild pain and the rest (12%) reported moderate pain; in the lidocaine group as well, most (58%) reported moderate pain and the rest (42%) reported mild pain. Saravanan et al. [38] found that, during flap elevation, only 8.62% (10) of the patients in the articaine group required re-anesthesia but all of the patients in the lidocaine group did. The comparison in the context of smooth extraction was statistically significant, with 91.38% of the patients having undergone smooth extraction in the articaine group compared with only 0.90% in the lidocaine group. Chandrasekaran et al. [43] reported that 49 of the 50 patients in the articaine group had successful extraction and in only one patient, the extraction was not possible while only two of the 50 patients in the lidocaine group had successful extractions. Phyo et al. [47], one of the studies that used the VAS to evaluate pain, reported that 86.67% (26) of the patients in the articaine group underwent tooth extractions without the need for a supplemental injection while 13.33% (four) did not. In the lidocaine group, 83.3% (25) of the patients underwent extraction without the need for supplemental injection while 16.7% (five) did not. In the study by Rayati et al. [48], pain was recorded subjectively as the answer “yes” or “no” when the patients were asked whether their extractions were painful; 36% of the patients (27) in the articaine group answered in the affirmative while 90.63% (58) in the lidocaine group answered in the affirmative.

Single Buccal Infiltration of 4% Articaine Compared With Double (Buccal and Palatal/Lingual) Infiltration of 4% Articaine

Four of the studies compared procedural pain during the extraction of permanent maxillary teeth between single buccal infiltration of 4% articaine and double (buccal and palatal/lingual) infiltration of 4% articaine ) Fan et al. [29], Uckan et al. [45], Sochenda et al. [46], and Iyengar et al. [51]. Procedural pain during extraction was evaluated subjectively using the VAS. Three of the four studies Fan et al. [29], Uckan et al. [45], Sochenda et al. [46], and Iyengar et al. [51] used other measures in addition to the VAS (the verbal rating scale [VRS], the FPS, and the Wong-Baker FPS, respectively). Fan et al. [29] found no significant difference in the VAS scores between the two types of injections for the removal of permanent maxillary teeth (P <0.05) and received no requests for additional palatal injections during either extraction, both of which were described as “acceptable” by the patients. Uckan et al. [45] analyzed the VAS and FPS scores of 23 patients who had undergone bilateral extractions using the student's t-test and reported the difference between permanent maxillary tooth removal with palatal injection (97.5%) and permanent maxillary tooth removal without palatal injection (96.8%) to be statistically significant (P < .05). Sochenda et al. [46] found that a buccal injection of 4% articaine and 1:100,000 epinephrine with no palatal injection had a success rate of 78.6%, while an 89.3% success rate was achieved with buccal and palatal infiltration injections of 4% articaine and 1:100,000 epinephrine, though the results were statistically insignificant for both groups (P-value of 0.083). Iyenger et al. [51] found that perfect anesthesia was achieved with buccal and palatal injections, with 100% of the patients in the control group reporting no pain prior to tooth extraction, but a buccal injection alone did not produce the expected effect, with only 26% of the patients who received this treatment reporting no pain. During probing or tissue separation, 18% of the patients complained of moderate or severe pain and 56% of mild pain, and the study group experienced higher pain levels, with 74% of the patients receiving palatal injections.

Single Buccal Articaine Compared With Double Lidocaine

Five of the studies compared procedural pain during the extraction of permanent maxillary teeth between single buccal infiltration of 4% articaine and double buccal and palatal/lingual infiltration of 2% lidocaine Somuri et al. [21], Luqman et al. [31], Bataineh et al. [35], Sandilya et al. [37], Al-Mahalawy et al. [53]. The five studies used a subjective score (the VAS), and two of them Somuri et al. [21], Luqman et al. [31] used in addition an objective score (the FPS). In Somuri et al. [21], 3 of the 15 patients in the articaine group experienced mild pain while none did in the lidocaine group, but the result was statistically insignificant. Luqman et al. [31] found that, in the articaine group, extraction was completed without the need for supplemental injection in (84% of the patients (18), and only 16% (16) needed a palatal injection, with the lowest VAS in the articaine group being recorded in the premolar area, but the results were statistically insignificant. Bataineh et al. [35] found that 74.5% of the patients (41) in the lidocaine group reported mild pain compared with 60% (30) in the articaine group; 25.5% of the patients (14) reported moderate pain in the lidocaine group compared with 34% (17) in the articaine group; and none of the patients reported severe pain in the lidocaine group compared with 6% (3) reporting it in the articaine group and requiring an additional palatal injection. Sandilya et al. [37] found that the VAS result was mainly in VAS-1, followed by VAS-0, for both groups and that, in the articaine group, six patients required a palatal injection compared with four patients in the lidocaine group who needed an extra palatal injection, while an extra buccal injection was required by five patients in the articaine group compared with four patients in the lidocaine group. However, none of these results were statistically significant. Al-Mahalawy et al. [53] found that none of the patients in either group needed an extra injection and that the VAS averaged 1.46 ± 0.80 in the articaine groups and 1.26 ± 0.82 in the lidocaine group, but these results were also statistically insignificant.

Single Buccal Infiltration of 4% Articaine 1:100:000 Compared With Single Buccal Infiltration of 4% Articaine 1:200:000 for the Extraction of Permanent Maxillary Teeth

Only the study by Lima Jr. et al. [23] compared the subjective pain between single buccal infiltration of 4% articaine 1:100:000 and single buccal infiltration of 4% articaine 1:200:000 for extracting impacted maxillary third molars with chronic pericoronitis without palatal injection. This study involved 30 patients between the ages of 15 and 46 years, half of whom received 4% articaine with 1: 100,000 epinephrine and half of whom received 4% articaine with 1: 200,000 epinephrine by buccal infiltration. The success rate was measured as the number of extractions performed without using supplemental palatal injections. The patients were instructed to raise their left hands to signal “stop.” The significance of the differences between the experimental groups was investigated using chi-square tests and residual analysis, and the significance level was found to be P <.05. GraphPad Prism software (San Diego, CA) served to conduct the statistical analysis. None of the patients in the 1: 100,000 epinephrine group reported pain, indicating that the treatment was 100% effective, while three of the patients (20%) in the 1: 200,000 epinephrine reported pain, indicating 80% effectiveness. Significant differences were observed between the groups (x2 = 3.84, P = .0143). The authors acknowledged limitations of the study relating to the data analysis owing to the subjectivity of the pain measurement method, the absence of the “gold standard” treatment for comparison, and the small sample size.

Single Buccal Infiltration of 4% Articaine with No Control

As discussed, the study by Bataineh et al. [28] was the only one in the sample to evaluate the procedural pain during the extraction of permanent maxillary teeth from patients who received single buccal infiltration of 4% articaine. Based on a subjective pain score (the VAS), 90.6% (87) of the patients underwent the procedures without the need for an additional palatal injection whereas 9.4% (nine) did need an additional palatal injection. Further, 90% of the patients categorized the pain as mild and less than expected for tooth extraction.

Limitations

This review was subject to certain limitations. To begin with, the studies by Bataineh and Al-Sabri [28], Uckan et al. [45], Sochenda et al. [46], and Iyengar et al. [51] did not clarify the risk of bias. Also, that by Bataineh et al. [35] involved the use of a higher concentration of epinephrine in the lidocaine group that, the author suggested, contributed to the higher success rate in the lidocaine group. The local anesthetic agents used by Chandrasekaran et al. [43] included articaine, bupivacaine, and lidocaine, but only the data relating to articaine and lidocaine were discussed in the present study.

## Conclusions

Sufficient analgesia induction with single buccal infiltration of 4% articaine is comparable to buccal and palatal infiltration of 2% lidocaine for the extraction of permanent maxillary teeth. The higher concentration of epinephrine can contribute to a higher success rate. Though the extraction of upper permanent maxillary teeth with only single buccal infiltration of 4% articaine is possible, the data remain insufficient to conclude whether this technique can replace the gold standard of buccal and palatal infiltration, so further investigation is needed to establish the conclusion.
